# Thanks to Reviewers 2019

**DOI:** 10.5334/pb.531

**Published:** 2020-01-24

**Authors:** Editorial Team

**Affiliations:** 1 Psychologica Belgica, BE

## Abstract

All manuscripts published in Psychologica Belgica have been assessed conscientiously and unselfishly by expert reviewers. The quality of our journal totally depends on their valuable and constructive criticisms to the authors. Both the editors and the authors highly appreciate the input and dedication of all our reviewers. Many thanks.

Tim Bal – Universiteit Gent

Larissa Barber – San Diego State University

Arjan Blokland – Maastricht University

Jean-Sébastien Boudrias – Université de Montréal

David Bourguignon – Université de Lorraine

Senne Braem – Vrije Universiteit Brussels

Lieven Brebels – KU Leuven

Tinne Buelens – KU Leuven

Gaëtane Caesens – UC Louvain

Jeroen Camps – KU Leuven

Dimitri Cauchie – Université de Mons

Alberto Crego – University of Santiago de Compostela

Anita Cservenka – Oregon State University

Jamie Cummins – Universiteit Gent

Benoit Dardenne – Université de Liège

Adélaïde de Heering – Université Libre de Bruxelles

Stéphanie Demoulin – UC Louvain

David Dignath – University of Freiburg

Martin Euwema – KU Leuven

Ludovic Ferrand – Université Clermont Auvergne

Sarah Galdiolo – UC Louvain

Amarendra Gandhi – KU Leuven

Matthew Grawitch – Saint Louis University

Michel Hansenne – Université de Liège

Alexandre Heeren – UC Louvain

Tom Heyman – KU Leuven

Graham Hitch – University of York

Sarah Kasran – Universiteit Gent

Glenn Kiekens – KU Leuven

Baptist Liefooghe – Universiteit Gent

Yara Mahfud – Université Paris Descartes

Randy McCarthy – Northern Illinois University

Loes Meeussen – KU Leuven

Catherine Monnier – Université Paul Valéry Montpellier 3

Tal Moran – Universiteit Gent

José Navarro – Universitat de Barcelona

Frédéric Nils – UC Louvain

Ricardo Pagán-Rodríguez – University of Malaga

Karin Proost – KU Leuven

Etienne Quertemont – Université de Liège

Isabel Raemdonck – UC Louvain

Arne Roets – Universiteit Gent

Tony Ross-Hellauer – Graz University of Technology

Alexander Savi – Vrije Universiteit Amsterdam

Dirk Smits – KU Leuven

Andromachi Spanouli – Tilburg University

Laurent Taskin – UC Louvain

Jon Tennant – University of Southern Denmark

Nicolas Van der Linden – Université Libre de Bruxelles

Wolf Van Paemel – KU Leuven

Marie-Anne Vanderhasselt – Universiteit Gent

Tim Vanhoomissen – Thomas More University of Applied Sciences

Vasilis Vasiliou – UC Louvain

Raquel Vazquez-Morejon – Universidad de Sevilla

Jenny Veldman – KU Leuven

Frederick Verbruggen – Universiteit Gent

Evy Woumans – UC Louvain

Melvin Yap – National University of Singapore

